# Effects of 5α-Dihydrotestosterone and 17β-Estradiol on the Mouse Ovarian Follicle Development and Oocyte Maturation

**DOI:** 10.1371/journal.pone.0099423

**Published:** 2014-06-09

**Authors:** Wataru Tarumi, Masanori T. Itoh, Nao Suzuki

**Affiliations:** 1 Department of Obstetrics and Gynecology, St. Marianna University School of Medicine, Kawasaki, Japan; 2 Department of Biology, College of Liberal Arts and Sciences, Tokyo Medical and Dental University, Ichikawa, Chiba, Japan; China Agricultural University, China

## Abstract

We have previously reported that androstenedione induces abnormalities of follicle development and oocyte maturation in the mouse ovary. In granulosa cells of the ovarian follicle, androstenedione is aromatized to 17β-estradiol (E_2_). To determine whether the androgen or estrogen acts directly on the follicle to induce the above-mentioned abnormalities, we compared the effects of a non-aromatizable androgen, 5α-dihydrotestosterone (DHT), with those of E_2_ on murine follicular development and oocyte maturation in a single follicle culture system. The high dose (10^−6^ M) of DHT prompted normal follicular development, and there was no effect on oocyte meiotic maturation after stimulation with human chorionic gonadotropin (hCG) and epidermal growth factor (EGF). In contrast, culture with the high dose (10^−6^ M) of E_2_ delayed follicular growth and also suppressed proliferation of granulosa cells and antrum formation. Furthermore, culture with E_2_ delayed or inhibited oocyte meiotic maturation, such as chromosome alignment on the metaphase plate and extrusion of the first polar body, after addition of hCG and EGF. In conclusion, these findings demonstrate that E_2_, but not DHT, induces abnormalities of follicular development, which leads to delay or inhibition of oocyte meiotic maturation.

## Introduction

In mammals, the processes of ovarian folliculogenesis and oogenesis are regulated by interaction between the oocyte and the surrounding somatic cells of the follicle, such as granulosa cells and theca cells [1]. In the ovary, oocytes are arrested in prophase of the first meiotic division, *i.e.,* at the germinal vesicle (GV) stage [2], [3]. When a fully grown GV stage oocyte in a large antral follicle is exposed to the surge of luteinizing hormone, the oocyte resumes meiosis and ovulation eventually occurs [4].

Ovarian steroid hormones, including androgens and estrogens, influence the processes of folliculogenesis and oogenesis through interaction with specific receptors [5], [6]. The effects of androgens, especially testosterone (T) and androstenedione, on folliculogenesis and oogenesis are controversial. T and androstenedione have been reported to increase the number of pyknotic granulosa cells and degenerating oocytes [7]–[9]. In addition, androstenedione inhibits the oocyte meiotic maturation, including spindle microtubule organization, alignment of chromosomes on the metaphase plate, and exclusion of the first polar body [9]. On the other hand, it has been demonstrated that T promotes growth during early folliculogenesis, since administration of T to rhesus monkeys significantly increases the number of preantral and small antral follicles, as well as stimulating the proliferation of granulosa and theca cells [10]. *In vitro* studies have shown that 5α-dihydrotestosterone (DHT) stimulates preantral follicle growth and granulosa cell mitosis in mice [11]. DHT also promotes the transition of primary follicles to secondary follicles in cattle [12] and improves follicular viability in humans [13].

The influence of estrogens on ovarian folliculogenesis and oogenesis is also not fully understood [14], [15]. It has been reported that treatment with estrogens, especially 17β-estradiol (E_2_), stimulates follicle growth and granulosa cell mitosis [16]. In addition, studies of hypophysectomized rats and mutant mice lacking follicle-stimulating hormone (FSH) or its receptor have shown that E_2_ and FSH exert a synergistic stimulatory effect on granulosa cell proliferation in preantral follicles [17]. In estrogen receptor (ER) β knockout mice, progression of follicles from the early antral to large antral stage is impaired, E_2_ production is decreased, and ovulation is also reduced, indicating that signaling via ERβ is necessary for both folliculogenesis and ovulation [18]–[20]. On the other hand, it has been reported that E_2_ has a marked influence on meiotic spindle organization and promotes multipolar spindle formation [21]. In addition, exposure to estrogen valerate induces the formation of follicular cysts that have thin layers of granulosa cells and lack oocytes [22].

Theca cells provide mainly androstenedione and T to granulosa cells [23], whereas granulosa cells convert androstenedione and T to E_2_ [24], [25]. Exposure to T or androstenedione increases E_2_ secretion from granulosa cells [9], [26]. Thus, it is possible that the effects of T and androstenedione on oocytes and the surrounding somatic cells of the follicle are mediated through E_2_. Accordingly, we compared the effects of the non-aromatizable androgen DHT and the representative estrogen E_2_ on follicular development and oocyte meiotic maturation in the present study using a murine single follicle culture system. We demonstrate that treatment with E_2_, but not DHT, induces morphologic and functional abnormalities in developing follicles. In addition, E_2_ delays or inhibits the oocyte meiotic maturation.

## Materials and Methods

These experiments were approved by the St. Marianna University School of Medicine Animal Care and Use Committee.

### Animals and harvesting of ovarian follicles

BDF1 female mice were maintained in a temperature- and light-controlled room (22°C; 14 h light/10 h dark with lights on at 0600). The animals had free access to food and water. Early preantral follicles were mechanically dissected from the ovaries with fine 26-G needles in α minimum essential medium (α-MEM) with Gluta MAX-I (Gibco BRL) supplemented with 5% heat-inactivated and charcoal-treated fetal bovine serum (FBS; Biowest, Nuaille, France). During the procedures, the medium was kept at 37°C. We selected early secondary follicles with a diameter of 100-130 µm (Type 3b from Pedersen classification) [27]. Twenty to 30 early secondary follicles were obtained from an ovary. The follicles were obtained from ovaries of three to five mice and pooled.

### Single follicle culture

Early secondary follicles were plated at one follicle per well in 96-well plates (BD BioCoat; BD Falcon) containing 75 µl/well of medium without a mineral oil overlay. Follicles with an intact basal membrane that showed no gaps between the oocyte and surrounding granulosa cells were selected by observation under an inverted microscope at ×400 magnification for use in these experiments. The follicles were randomly divided into control and experimental groups (n = 40 per group). The culture medium was α-MEM plus Gluta MAX-I containing 5% FBS, 0.5% gentamicin, 5 mg/ml insulin, 5 mg/ml transferrin, 5 ng/ml selenium, and 10 mIU/ml human FSH. Gentamicin, insulin, transferrin, and selenium were obtained from Gibco BRL, while human FSH (Follistim) was obtained from Merck & Co. (Whitehouse Station, NJ, USA). Follicles were cultured in medium containing 10^−10^, 10^−8^, or 10^−6^ M DHT (0.02905–290.5 ng/ml) or E_2_ (0.02724–272.4 ng/ml) obtained from Sigma-Aldrich Co. (St. Louis, MO, USA), while control follicles were cultured in the vehicle alone (0.01% dimethyl sulfoxide). To assess the growth of each follicle, two perpendicular diameters were measured using a calibrated ocular micrometer at ×200 magnification and the oocyte diameter was also measured. Viable follicles were defined as those that retained an oocyte completely embedded within the granulosa cell mass, and the survival rate was expressed as a percentage of all plated follicles. The follicles were cultured for 13 days at 37°C under 5% CO_2_ in air. Every 4 days, 30 µl of the medium was exchanged.

### Induction of oocyte maturation

To induce the maturation of oocytes at the GV stage, 1.5 IU/ml of human chorionic gonadotropin (hCG; Sigma-Aldrich Co.) and 5 ng/ml of human epidermal growth factor (EGF; Upstate, Temecula, CA, USA) were added to the medium on day 13 of culture. After 16 h, cumulus cells surrounding oocyte were removed by pipetting, and then oocyte meiotic maturation was assessed by detection of GV breakdown (GVBD), which is an indicator of the resumption of meiosis, and by the presence of the first polar body (metaphase II stage; MII). The process of spindle formation was also observed.

### Progesterone (P_4_) assay

The concentrations of P_4_ in the spent medium were measured with ELISA kit (Neogen, St. Joseph, MI, USA). The intra-assay coefficient of variation was 3.6%, the interassay coefficient of variation was 6.0%, and the sensitivity was 0.4 ng/ml.

### Immunofluorescence

For immunofluorescence, oocytes were fixed in 1% formaldehyde at room temperature for at least 30 min. After washing with 0.5% Triton X-100 in phosphate-buffered saline (PBS, pH 7.4) three times for 15 min each, oocytes were permeabilized overnight at 4°C with 0.01% Triton X-100 in PBS. Then the oocytes were double-stained to visualize microtubules and DNA. Briefly, oocytes were incubated with fluorescein isothiocyanate-conjugated mouse anti-α-tubulin antibody (Sigma-Aldrich Co., 1∶200 at dilution) overnight at 4°C. After washing, DNA was stained with 10 µg/ml propidium iodide (Dojindo Laboratories, Kumamoto, Japan), and the oocytes were mounted on glass slides for observation with a laser-scanning Zeiss LSM510 confocal microscope. No staining was apparent when the primary antibody was omitted.

### Statistical analysis

All experiments were independently replicated at least twice. Quantitative values are presented as the mean ± SEM. Data were tested for homogeneity of variance using Bartlett's test and Levene's test. The follicle diameter, oocyte diameter, follicle survival rate, and P_4_ levels were compared by two-way repeated measures analysis of variance with a post hoc Tukey's test. Data on oocyte maturation were analyzed by Dunnett's post hoc test for comparison of the effect of DHT or E_2_ versus the control. In all analyses, *P*<0.05 was considered to indicate statistical significance.

## Results

### Follicular growth and viability

**Figure 1:**
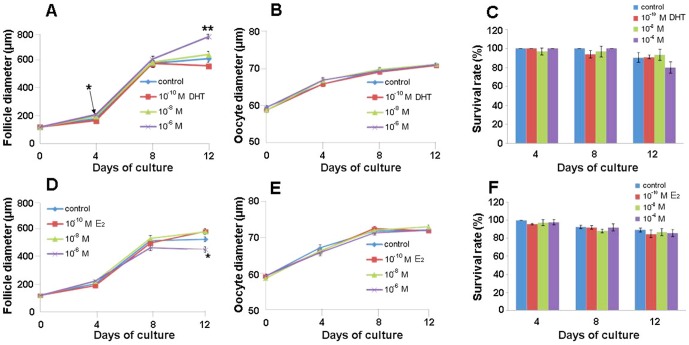
Changes in the diameters of ovarian follicles and oocytes and in survival rates of follicles. Follicles were cultured individually for 12 days in the presence of DHT (A-C) and E_2_ (D-F). (A), (B), (D) and (E) are representatives of four independent experiments (n = 40 follicles per group). (C) and (F) show the results of four independent experiments, and values are expressed as the mean ± SEM. *P<0.05 vs. control.

Both the follicular and oocyte diameters increased during culture in all groups (Fig. 1A, B, D and E). On days 4 and 12 of culture, the follicular diameter was significantly larger with 10^−6^ M DHT treatment than with vehicle treatment (control) (Fig. 1A). In contrast, 10^−6^ M E_2_ significantly inhibited follicular growth on day 12 (Fig. 1D). Neither DHT nor E_2_ had any effect on oocyte growth (Fig. 1B and E) or on follicle survival rate (Fig. 1C and F).

### Follicle morphology

**Figure 2:**
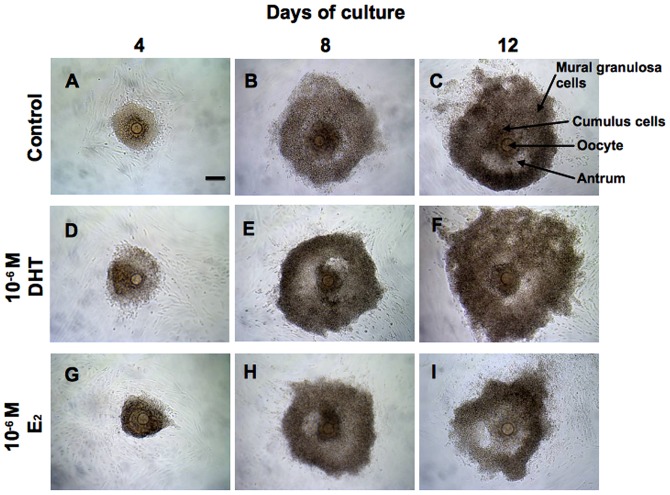
Representative ovarian follicles cultured for 4, 8, or 12 days with the vehicle only (control) (A-C), 10^−6^ M DHT (D-F), or 10^−6^ M E_2_ (G-I). Scale bar in (A), 100 µm. The scale bar is applicable to all panels.

When cultured with 10^−6^ M DHT or the vehicle only (control) for 12 days, the follicles grew to form thick layers of mural granulosa cells and large antral cavity (Fig. 2A-F). In contrast, most of the follicles treated with 10^−6^ M E_2_ showed abnormal morphology on day 12 with thin layers of mural granulosa cells than in control and DHT-treated follicles (Fig. 2I).

### Oocyte maturation and follicular P_4_ secretion after stimulation with hCG and EGF

**Figure 3:**
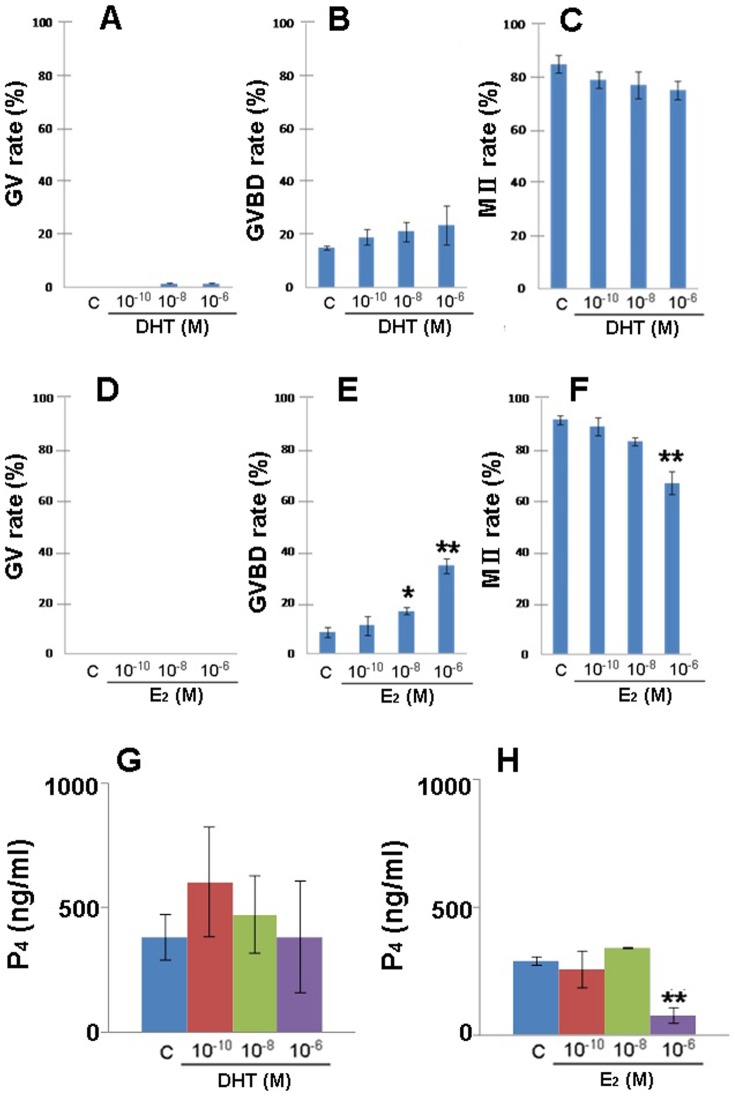
Oocyte meiotic maturation and P_4_ secretion by follicles at 16 h after stimulation with hCG and EGF. Follicles were cultured individually for 12 days in the presence of DHT, E_2_, or vehicle only (control; C), and then were stimulated with hCG and EGF for 16 h. During this period, normal oocytes advanced from GV stage through GVBD to MII with exclusion of the first polar body. The percentage of oocytes at each stage was calculated for follicles cultured with DHT (A-C) or with E_2_ (D-F).(G and H) P_4_ secretion from follicles stimulated for 16 h with hCG and EGF. All values are expressed as the mean ± SEM (n = 20 per group). *P<0.05 vs. control.

Follicles were cultured for 12 days, and then were treated with hCG and EGF. After 16 h of this treatment, the progress of oocyte meiotic maturation was assessed (Fig. 3A-F), and levels of P_4_ in the culture medium were determined (Fig. 3G and H). With regard to the percentages of GV, GVBD, and MII oocytes, there were no significant differences between the DHT group and the control group (Fig. 3A-C). After 16 h of incubation with hCG and EGF, 75 to 80% of the oocytes in the DHT group reached the MII stage with exclusion of the first polar body (Fig. 3C). These findings indicate that DHT does not affect the progression of oocyte meiotic maturation. On the other hand, the percentage of GVBD oocytes showed a significant increase in follicles treated with 10^−8^ and 10^−6^ M E_2_ (Fig. 3E). In 10^−6^ M E_2_-treated follicles, the percentage of MII oocytes with exclusion of the first polar body was significantly decreased (Fig. 3F). These results indicate that E_2_ treatment blocks the progression from GVBD to MII in oocytes.

There was no significance in P_4_ secretion between DHT-treated and control follicles (Fig. 3G). In contrast, P_4_ secretion from 10^−6^ M E_2_-treated follicles was significantly lower than that by control follicles (Fig. 3H).

### Spindle formation by oocytes after hCG and EGF stimulation

**Figure 4:**
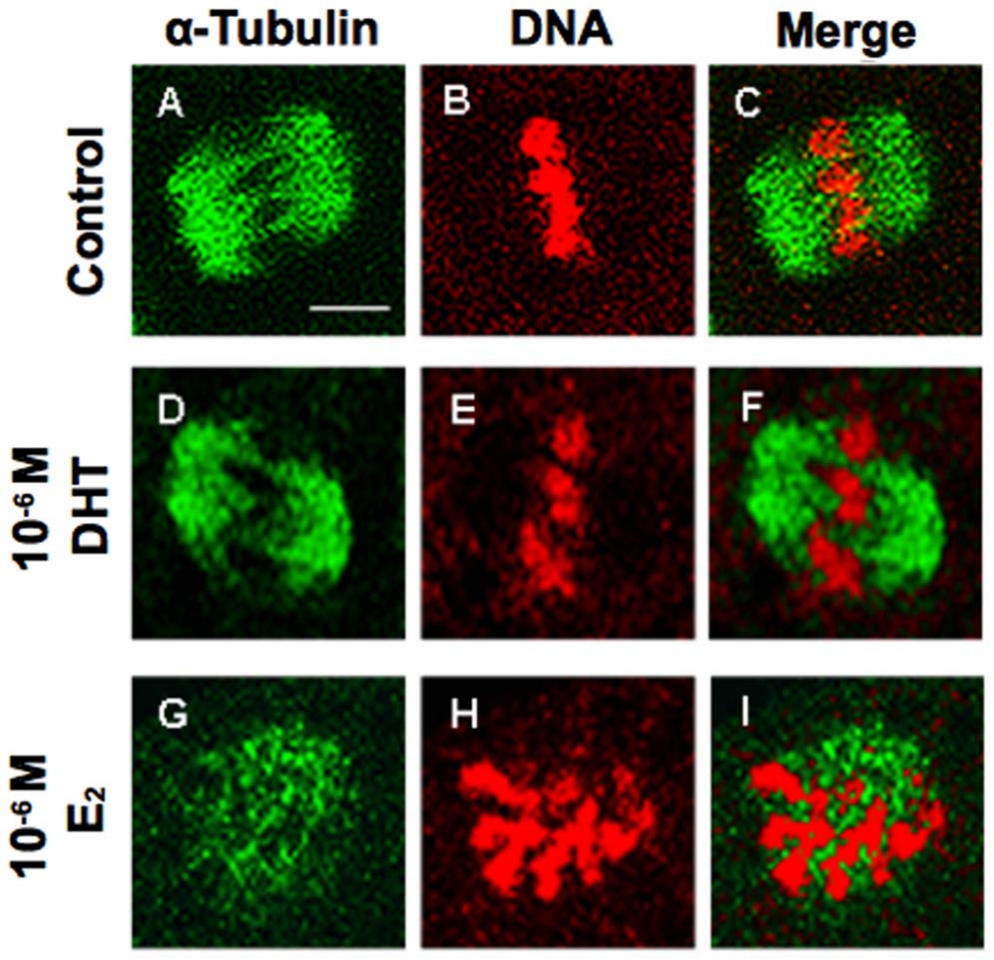
Oocyte spindle formation after 16 Follicles were cultured individually for 12 days with DHT, E_2_ or vehicle only (control), and then were stimulated with hCG and EGF. Spindle fibers were detected by immunofluorescence for α-tubulin (green), while DNA was stained with propidium iodide (red). Scale bar in (A), 20 µm. The scale bar is applicable to all panels.

We examined spindle formation in oocytes histochemically after 16 h of stimulation with hCG and EGF. In follicles treated with 10^−6^ M DHT, 75% of oocytes showed morphologically normal spindle assembly, chromosome alignment, and chromosome segregation, as seen in control follicles (Fig. 4D-F). In 10^−6^ M E_2_-treated follicles, however, 35% of oocytes showed inhibition of spindle formation, with changes such as abnormal spindle assembly and abnormal chromosome alignment (Fig. 4G-I).

**Figure 5:**
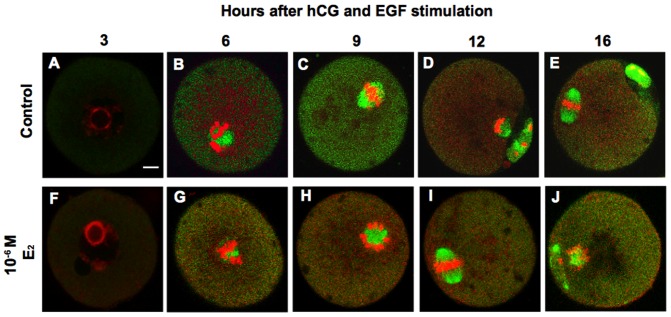
Time course of oocyte spindle formation after stimulation with hCG and EGF. Follicles were cultured individually for 12 days with the vehicle only (control) or 10^−6^ M E_2_, and then were stimulated with hCG and EGF. Spindle fibers were detected by immunofluorescence for α-tubulin (green), while DNA was stained with propidium iodide (red). The upper panels show oocytes from control follicles, while the lower panels show oocytes from follicles cultured with 10^−6^ M E_2_. Scale bar in (A), 10 µm. The scale bar is applicable to all panels.

We also examined the time course of spindle formation in oocytes from E_2_-treated follicles and control follicles after hCG/EGF stimulation. After 3 and 6 h of stimulation, spindle formation by all oocytes from follicles cultured with 10^−6^ M E_2_ seemed to be normal in comparison with oocytes from control follicles (Fig. 5A, B, F and G). In all oocytes from control and E_2_-treated follicles, GVBD took place after 3–6 h of hCG/EGF stimulation (Fig. 5A, B, F and G). After 16 h of stimulation, 85–95% of the control oocytes had reached the MII stage with exclusion of the first polar body (Fig. 5E). In contrast, 30–40% of oocytes from 10^−6^ M E_2_-treated follicles showed inhibition or delay of spindle formation, including abnormality of chromosome alignment and delayed exclusion of the first polar body (Fig. 5H-J).

## Discussion

In the previous study using a single follicle culture system, we demonstrated that androstenedione induced morphologic and functional abnormalities of developing mouse follicles, and impaired oocyte meiotic maturation [9]; androstenedione treatment reduced follicle viability and led to the formation of abnormal follicles, including those with misshapen oocytes. Moreover, when androstenedione-treated follicles were stimulated with hCG and EGF, spindle microtubule organization, chromosome alignment on the metaphase plate, and exclusion of the first polar body were inhibited in oocytes. Granulosa cells of the ovarian follicle aromatize androstenedione to E_2_ by the sequential actions of 17β-hydroxysteroid dehydrogenase and cytochrome P450 aromatase [25]. Thus, it is possible that E_2_ derived from androstenedione acts on follicular somatic cells and/or oocytes to induce such morphologic and functional abnormalities. To determine whether the androgen or estrogen was responsible for these abnormalities, we compared the effects of the non-aromatizable androgen DHT with those of E_2_ on follicular development and oocyte meiotic maturation in the present study. Our results indicated that DHT stimulated normal development of ovarian follicles. It has been reported that DHT stimulates preantral follicle growth and granulosa cell mitosis in mice [11], as well as promoting the transition of primary follicles to secondary follicles in cattle [12] and increasing follicular viability in humans [13]. In addition, it has been reported that androgen receptors are mainly expressed by oocytes and granulosa cells in the ovary [28].

In contrast to the effects of DHT, the present study showed that E_2_ treatment prevented follicle growth, as well as decreasing P_4_ production after hCG/EGF stimulation. Moreover, treatment of follicles with E_2_, but not DHT, inhibited or delayed spindle formation (including chromosome alignment on the metaphase plate and first polar body exclusion) after hCG/EGF stimulation. It has been reported that E_2_ treatment delays cell cycle progression by acting on centrosomal proteins, as well as microtubules to a lesser extent, leading to abnormal spindle formation and chromosome non-disjunction [29]. In follicles treated with E_2_, a multipolar spindle is the most frequent abnormality [21]. In addition, exposure to estrogen valerate induces the formation of follicular cysts with thin layers of granulosa cells that lack oocytes [22]. These reports and our findings demonstrate that E_2_, but not DHT, induces morphologic and functional abnormalities of developing follicles, which results in the impairment of oocyte meiotic maturation. The treatment of oocytes with hCG/EGF induces meiotic resumption specially from GV to GVBD [30], [31]. In the present study, the high dose (10^−6^ M) of E_2_ blocked the progression from GVBD to MII in oocytes. Thus, the high dose of E_2_ may not block the action of hCG/EGF on oocytes.

The effects of E_2_ on follicular development and oocyte maturation are mediated through interaction with specific ERs [32], [33]. Two ERs, ERα and ERβ, are known to transduce estrogenic signals [34], [35]. ERα is expressed in cumulus cells, germinal epithelium, interstitial cells and thecal cells, while ERβ is expressed in oocytes, cumulus cells, and granulosa cells in primary, secondary, and mature follicles [6], [19], [32]. In ERβ-knockout mice, but not ERα-knockout mice, follicles shows significantly less progression from the early antral to large antral stage, E_2_ production is decreased, and ovulation is reduced, indicating that signaling via ERβ is necessary for both folliculogenesis and ovulation [18]–[20].

The interaction of DHT and ERs has been studied for a long time [36]. It is known that the binding affinity of DHT to ERα is tremendously (1800 times) lower than that of E_2_ and it to ERβ has low 600 times lower [37]. DHT is not metabolized to estrogens [41]. In the present study, the high dose (10^−6^ M) of DHT prompted normal follicle development, but E_2_ delayed follicular growth. In addition, 10^−6^ M DHT did not affect the spindle formation of oocyte, but that E_2_ did at the highest dose. The previous and present studies demonstrate the binding affinity of DHT and ERs much lower than that of E_2_.

In the present study, P_4_ production after hCG/EGF stimulation was reduced by treatment of follicles with 10^−6^ M E_2_. P_4_ receptors are known to be expressed by oocytes and granulosa cells [38], [39], and P_4_ has been reported to promote oocyte maturation in primates [40], swine [41], and cattle [42]. Therefore, it is suggested that a decrease of P_4_ secretion from follicles leads to disturbance of oocyte maturation.

In conclusion, E_2_ acts on oocytes and granulosa cells in developing follicles to induce various morphologic and functional abnormalities of these cells. E_2_ also delays or inhibits oocyte meiotic maturation. In contrast, the non-aromatizable androgen DHT does not induce any of these abnormalities. These findings suggest that the inhibitory effects of androstenedione and T on follicular development and oocyte meiotic maturation are mediated through E_2_ that is the metabolite of androstenedione and T.
